# Hematological profiles of malaria-infected patients in an endemic area of Peru

**DOI:** 10.17843/rpmesp.2022.393.11908

**Published:** 2022-09-30

**Authors:** Viviana Pinedo-Cancino, Katty M. Arista, Andree Valle-Campos, Rafael Saavedra-Langer, Cristian Roca, José-Manuel Ramos-Rincón, Maritza Calderón, Oralee H. Branch

**Affiliations:** 1 Laboratory for Research on Natural Antiparasitic Products of the Amazon, Natural Resources Research Center, Universidad Nacional de la Amazonía Peruana, Iquitos, Perú. Universidad Nacional de la Amazonía Peruana Laboratory for Research on Natural Antiparasitic Products of the Amazon Natural Resources Research Center Universidad Nacional de la Amazonía Peruana Iquitos Peru; 2 Faculty of Human Medicine, Universidad Nacional de la Amazonía Peruana, Iquitos, Peru. Universidad Nacional de la Amazonía Peruana Faculty of Human Medicine Universidad Nacional de la Amazonía Peruana Iquitos Peru; 3 Department of Biological Sciences. Universidad Nacional Mayor de San Marcos, Lima, Peru. Universidad Nacional Mayor de San Marcos Department of Biological Sciences Universidad Nacional Mayor de San Marcos Lima Peru; 4 Department of Biochemistry and Immunology, Institute of Biological Sciences, Universidad Federal de Minas Gerais, Belo Horizonte, Minas Gerais, Brazil. Universidade Federal de Minas Gerais Department of Biochemistry and Immunology Institute of Biological Sciences Universidad Federal de Minas Gerais Belo Horizonte Minas Gerais Brazil; 5 Department of Microbiology and Immunology, University of North Carolina, Chapel Hill, United States. Department of Microbiology and Immunology University of North Carolina Chapel Hill Estados Unidos; 6 Department of Internal Medicine, Hospital General Universitario de Alicante-ISABIAL, Alicante, Spain. Department of Internal Medicine Hospital General Universitario de Alicante-ISABIAL Alicante España; 7 Department of Clinical Medicine, Universidad Miguel Hernández de Elche, Alicante, Spain. Universidad Miguel Hernández de Elche Department of Clinical Medicine Universidad Miguel Hernández de Elche Alicante Spain; 8 Infectious Diseases Research Laboratory, Universidad Peruana Cayetano Heredia, Lima, Peru. Universidad Peruana Cayetano Heredia Infectious Diseases Research Laboratory Universidad Peruana Cayetano Heredia Lima Peru; 9 Lark Health, Clinical studies, Mountain View, California, United States. Mountain View Lark Health, Clinical studies Mountain View California USA

**Keywords:** *Plasmodium*, Blood Cell Count, Platelets, Tropical Disease, Peruvian Amazon, Parasites

## Abstract

**Objectives.:**

To evaluate the variation of hematological profiles of patients infected with uncomplicated Plasmodium vivax (Pv) and P. falciparum (Pf) malaria before, during and after treatment in a population of the Loreto region.

**Materials and methods.:**

This study was conducted between 2010 and 2012, in Zungarococha (Iquitos). The 425 participants had three visits (visit 1-day 0-before treatment, visit 2-day 7-during treatment, visit 3-day 28-after treatment), complete blood count, microscopic and molecular diagnosis (PCR).

**Results.:**

At the first visit, 93 (21.9%) participants were found positive for Pv and 34 (8.0%) for Pf. All positives showed a reduction in hematocrit, white blood cell count (WBC), ablated and segmented neutrophils, eosinophils and platelets (p<0.001) compared to the negative group. A higher percentage of ablated neutrophils was found in Pf and segmented neutrophils in Pv compared to the negative group. Variations in hematological profiles were observed after treatment for both species; ablated neutrophils decreased, platelets increased, eosinophils increased at day 7 and declined at day 28, hematocrit and segmented neutrophils decreased at day 7 and normalized at day 28. Interspecies differences over time showed a bigger daily decrease in ablated neutrophils in Pv-infected when compared to Pf.

**Conclusions.:**

The hematological profile in uncomplicated malaria-positive patients varies over time during and after treatment. These are indicators of disease progression and help in the therapeutic surveillance of Plasmodium-infected patients.

## INTRODUCTION

Malaria has wreaked havoc on mankind since ancient times and remains a priority public health problem in tropical and subtropical regions of the world. There are currently five species of parasites of the genus *Plasmodium* that cause disease in humans, which are transmitted by the bite of mosquitoes of the genus *Anopheles*, with *Plasmodium vivax* (Pv) and *Plasmodium falciparum* (Pf) being the most dangerous and frequent ^1^.

In 2020, there were 241 million malaria cases and 627,000 deaths worldwide, with an increase of 14 million cases and 69,000 deaths from the 2019 figures ^2^; with a sharp increase in malaria cases and deaths globally, despite the effort deployed by malaria control programs to achieve malaria elimination in endemic countries, which were affected by the interruptions in health services during the COVID-19 pandemic ^3^.

In Peru, malaria permanently threatens the health of the population, mainly in the department of Loreto (capital, Iquitos) where 90% of cases are concentrated, with a predominance of Pv followed by Pf ^4^ ^-^ ^6^. This department is classified as an endemic region with low transmission among rural and peri-urban communities, although there are high foci of transmission in some parts of Loreto ^7^. The clinical characteristics of malaria depend on the species and range from short febrile episodes to severe systemic complications and death ^3^ ^,^ ^8^.

The change in leukocyte parameters is the most common complication found in malarial patients and plays an important role in the clinical severity of the disease and mortality ^9^. Malaria with severe organ failure or blood abnormalities is usually associated with Pf ^3^ ^,^ ^10^. In addition to severe anemia, there are other alterations such as thrombocytopenia or reduction in the number of platelets, leukocytosis or leukopenia, hemoglobinuria, cardiovascular collapse and shock, acute kidney injury, metabolic acidosis, and hypoglycemia ^11^ ^-^ ^13^. In contrast, mild malaria, with benign forms, is related to Pv, but is also capable of producing severe anemia, shock, lung injury, acute renal failure, and cerebral malaria ^12^ ^,^ ^13^.

Early diagnosis is important for prompt and timely treatment and disease management ^1^ ^-^ ^3^. However, knowledge of the hematological characteristics of malaria is limited to Pf cases from African and Asian regions ^14^ ^,^ ^15^, and little or nothing is known about the leukocyte characteristics of Pv in endemic populations of Loreto; its relationship as a tool for hematological diagnosis could allow a more adequate management of the possible complications of malaria. This study evaluated the variation of hematological profiles before, during and after treatment of patients infected with uncomplicated *Plasmodium vivax* (Pv) and *P. falciparum* (Pf) malaria in a population of the Loreto region, comparing it with healthy individuals from the same area for its potential use as predictors of disease progression and treatment efficacy.

**KEY MESSAGES**

Motivation for the study: to determine if there are hematological variations during uncomplicated malaria infection before, during and after treatment.

Main findings: variations in hematological profiles do occur in patients infected with *Plasmodium*, which normalize after treatment and are different according to the species.

Implications: the prediction of these variations could allow establishing early therapeutic interventions in order to prevent complications.

## MATERIALS AND METHODS

### Type of study, study population and data collection

A longitudinal descriptive study was conducted with samples collected between 2010 and 2012, from a cohort study of the Immunology and Genetics of Malaria in the Amazon Project, developed in the community of Zungarococha, which groups four villages: Zungarocoha, Puerto Almendra, Nina Rumi and Llanchama (Iquitos, Peru), where Pv and Pf infections are often asymptomatic with low parasite density ^16^. Participants who entered the project and who had signed consent and/or assent and accepted the future use of their samples were included in the study; all patients who traveled during the course of the visits were excluded. All participants were visited three times: visit 1, day 0 (before treatment); visit 2, day 7 (during treatment); visit 3, day 28 (after treatment).

The sample collection process was passive and active, as detailed by Branch, *et al* ^16^. For each participant, we obtained a blood sample by a fingerstick blood test collecting 500 µL in 1.5 mL microtubes with 40 µL of 0.05 M EDTA or a venous puncture using 3 mL Vacutainer tubes with EDTA K2. For hematocrit, we used capillary tubes of 75 mm in length and about 1.5 mm in diameter; in addition, the blood smear was carried out on 2 plates for hemogram and microscopic diagnosis of malaria. Erythrocytes were separated from plasma by centrifugation at 1200 rpm for 10 min and frozen at -20 °C until DNA extraction.

The hematological analysis was carried out manually. The hematocrit was measured as the volume of packed cells after centrifugation at 1200 rpm for 5 min, using a millimeter ruler that measured the columns that formed inside the capillaries. The percentage corresponding to the erythrocyte column was calculated with a rule of three. The smear was stained with Wright solution for one minute at room temperature. Subsequently, water was added in equal parts to the dye and left for 10 min, then it was washed, and dried in order to carry out the differential count of white blood cells up to 100 cells with the immersion objective (100x) of the microscope. Two different technicians worked on the hematological analysis of the smears and they only knew about the results of the samples they analyzed.

The blood smear for malaria diagnosis was stained with Giemsa stain and observed under the microscope with immersion oil. In order to establish the parasite density, we used the cross-matching method, and the parasites per microliter of blood method ^16^.

*Plasmodium* DNA was extracted from 200 µL of erythrocytes using the QIAamp DNA blood minikit (Qiagen, Hilden, Germany). The DNA samples were then stored at -20 °C until amplification. For molecular diagnosis, the semi nested multiplex malaria PCR (SnM-PCR) technique was used conventionally, with external and internal amplification and 1.5% agarose gel electrophoresis for identification of *Plasmodium* species ^17^.

### Statistical analysis

Data analysis was carried out with the statistical package Stata v.15.0 (StataCorp, College Station, TX, USA). For comparisons of demographic and laboratory characteristics between the group of *Plasmodium* sp-positive participants and the group of negative participants (participants without malaria), we used Chi-square and Kruskal-Wallis tests, with a 95% confidence interval, and a significant p <0.05. The Chi-square test was chosen to test the hypotheses of equality of proportions for categorical variables, and the Kruskal-Wallis test was used to test the hypotheses of equality of medians for continuous numerical variables with non-normal distribution.

The criteria for constructing the linear models were the following: All hematological profile variables were included in a generalized linear model with identity link adjusted for age and sex, in order to quantify the variations of the hematological profiles of each *Plasmodium* species versus the negative group at visit 1 (corresponding to day 0 of the study). The coefficients of the model were interpreted as the average of the magnitude of change, in their respective unit of measurement, of the hematological indicator in a given *Plasmodium* species compared to the negative group. The analysis of variations in the hematological profile over time: visit 2-day 7 (during treatment) and visit 3-day 28 (after treatment) with *Plasmodium* species, we used a linear regression model with Gaussian distribution and identity-linked function for each hematological variable as the outcome. Time was inserted into two separate models: i) assuming time-outcome linearity and ii) day of each visit. In Model 1, time was considered a continuous variable, using the time interval in days, between the initial measurement (visit 1, day 0, before treatment) and the follow-up date (visit 2, day 7, during treatment and visit 3, day 28, after treatment). In Model 2, time was considered a categorical variable according to visit, where the initial measurement was day 0 (visit 1), day 7 (visit 2) and day 28 (visit 3). Each participant had three measurements for each hematologic variable. The time invariant exposure variable was *Plasmodium* species, which was adjusted for age and sex. The analysis is available on GitHub (https://github.com/avallecam/hemogr/) and Zenodo (https://doi.org/10.5281/zenodo.4014204).

### Ethical Aspects

This research was conducted following the principles outlined in the Declaration of Helsinki and participation was voluntary. This study was approved by the Ethics Committee of New York University (NYU Institutional Review Board [IRB] APROVAL NO. 08-982) and the Peruvian Ministry of Health and the Ethics Committee of the Instituto Nacional de Salud. The individuals who volunteered to participate signed an informed consent form after receiving information about the study.

## RESULTS

Between the years 2010 and 2012, 425 participants aged from 10 to 44 years were evaluated; 236 were female and 189 were male. 

All participants were confirmed to have malaria by microscopy, which resulted in 93 (21.9%) positive for Pv, 34 (8.0%) positive for Pf and 298 (70.1%) negative for malaria. All microscopy-positive results and 99 participants randomly selected from the 298 microscopy-negative were evaluated by PCR to confirm the results.

As shown in Table 1, the baseline hematological indicators such as hematocrit, blood cell count (RGB), banded neutrophils, segmented neutrophils, eosinophils, and platelets were statistically different (p < 0.001) among participants infected with uncomplicated malaria compared to the negative group (participants without malaria). Monocytes and lymphocytes in the studied groups were not statistically different from each other.

**Table 1:** Baseline distribution (corresponding to visit 1 on day 0 of the study) of demographic characteristics and hematologic profile among negative and positive participants by *Plasmodium* species.

Variable	Negative		*P. vivax*		*P. falciparum*		p-value ^b^
	N=298		N=93		N=34		
	Median (IQR)		Median (IQR)		Median (IQR)		
Age (years)	28	(17.0 - 42.8)	17	(10.0 - 39.0)	24,5	(14,2 - 44,2)	0,001
Sex ^a^							0.082 ^c^
Female	175	(58.7)	47	(50.5)	14	(41.2)	
Male	123	(41.3)	46	(49.5)	20	(58.8)	
Hematocrit (%)	42	(39.0 - 44.0)	39	(36.0 - 42.0)	39,5	(37.2 - 41.8)	<0.001
Leucocytes (10³/mm³)	6.2	(5.9 - 7.0)	5.8	(5.1 - 6.0)	5.9	(5.0 - 6.2)	<0.001
Banded (%)	0	(0.0 - 0.0)	2	(0.0 - 4.0)	4.5	(0.0 - 7.5)	<0.001
Neutrophils (%)	58	(50.2 - 60.0)	60	(56.0 - 62.0)	57,5	(54.0 - 60.0)	0.009
Eosinophiles (%)	9	(5.0 - 12.0)	5	(3.0 - 8.0)	5	(4.0 - 8.8)	<0.001
Lymphocytes (%)	34	(29.2 - 38.8)	32	(30.0 - 35.0)	30	(27.0 - 36.0)	0.075
Platelets (10⁴/mm³)	17.3	(16.0 - 19.7)	14.9	(13.6 - 17.1)	14.8	(13.2 - 17.4)	<0.001
Monocytes (%) ^a^							0.283 ^c^
0	289	(97.0)	91	(97.8)	32	(94.1)	
1	5	(1.7)	2	(2.2)	0	(0.0)	
2	3	(1.0)	0	(0.0)	2	(5.9)	
3	1	(0.3)	0	(0.0)	0	(0.0)	
Basophiles (%) ^a^							0.159 ^c^
0	279	(93.6)	93	(100.0)	34	(100.0)	
1	15	(5.0)	0	(0.0)	0	(0.0)	
2	3	(1.0)	0	(0.0)	0	(0.0)	
3	1	(0.3)	0	(0.0)	0	(0.0)	

IQR: interquartile range.

a Number and percentage, N (%).

b Kruskal-Wallis test

c Chi-square test

**Figure 1:**
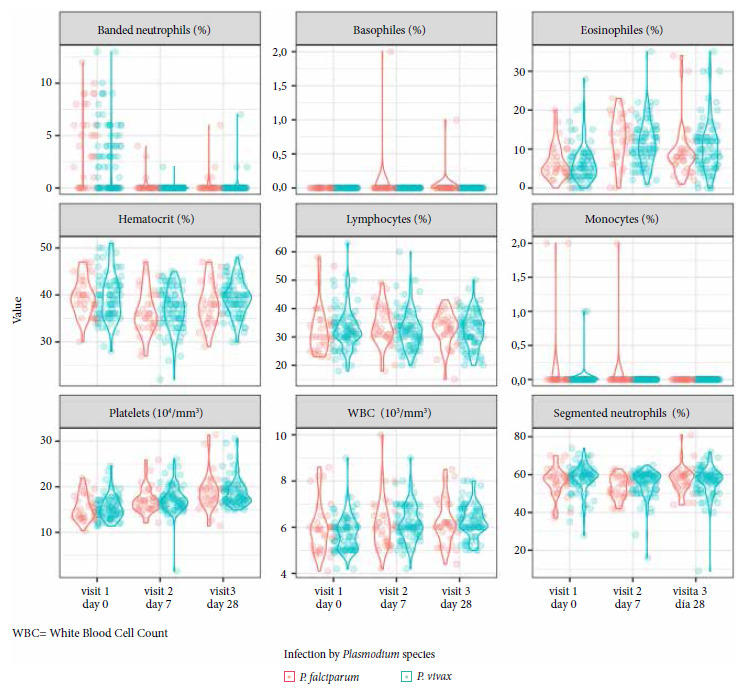
Hematological profiles by day of visit and *Plasmodium* species.

For visit 1 (day 0 of the study), analyses of the generalized linear model with identity linkage adjusted for age and gender conducted to quantify the differences between participants infected with *P. vivax* and *P. falciparum* (Figure 1) compared to the negative group (Table 2), showed that participants infected with Pv and Pf obtained low percentages for hematocrit (Pv: -2.3%, Pf: -2.7%), RGB (Pv: -0.6%, Pf: -0.5%), eosinophils (Pv: -3.6%, Pf: -3.2%) and platelets (Pv: -2.8%, Pf: -2.6% x 104 platelets/mm^3^) compared to the negative group (participants without malaria). On the contrary, malaria-infected participants obtained high percentages of banded neutrophils (Pv: 2.1%, Pf: 3.7%) compared to the negative group. We found a different pattern only in segmented neutrophils, where only those infected with Pv had a higher percentage (2.7%) compared to Pf and the negative group.

**Table 2:** Hematologic differences at visit 1 (day 0 of the study) between negative and positive participants by *Plasmodium* species in a multiple regression analysis.

Variable	Negative		*P. vivax*			*P. falciparum*		
	Median	(IQR)	Coef.	(95% CI)	p-value	Coef.	(95% CI)	p-value
Hematocrit (%)	42.0	(39.0 - 44.0)	-2.26	(-3.38 - -1.13)	<0.001	-2.71	(-4.36 - -1.06)	0.001
WBC (10³/mm³)	6.2	(5.9 - 7.0)	-0.64	(-0.84 - -0.44)	<0.001	-0.46	(-0.78 - -0.15)	0.004
Banded (%)	0.0	(0.0 - 0.0)	2.11	(1.64 - 2.59)	<0.001	3.70	(3.05 - 4.34)	<0.001
Segmented (%)	58.0	(50.2 - 60.0)	2.71	(1.00 - 4.43)	0.002	0.75	(-1.83 - 3.33)	0.568
Eosinophiles (%)	9.0	(5.0 - 12.0)	-3.58	(-4.97 - -2.19)	<0.001	-3.21	(-5.37 - -1.04)	0.004
Lymphocytes (%)	34.0	(29.2 - 38.8)	-1.13	(-2.93 - 0.68)	0.221	-1.22	(-4.02 - 1.57)	0.391
Platelets (10⁴/mm³)	17.3	(16.0 - 19.7)	-2.77	(-3.48 - -2.07)	<0.001	-2.62	(-3.72 - -1.52)	<0.001
Monocytes (%)	0.0	(0.0 - 0.0)	-0.04	(-0.10 - 0.03)	0.264	0.06	(-0.05 - 0.18)	0.274
Basophiles (%)	0.0	(0.0 - 0.0)	-0.08	(-0.15 - -0.01)	0.026	-0.08	(-0.20 - 0.03)	0.170

IQR: interquartile range, WBC: white blood cell count.

All *Plasmodium* species ratios were calculated in comparison to the group of negative participants and positive participants by Plasmodium species

The analysis of variations in the hematologic profile over time compares visit 2, day 7 (during treatment) and visit 3, day 28 (after treatment) with visit 1, day 0 and *Plasmodium* species. An increasing and decreasing continuous linear relationship between time and outcome was found in model 1 (Table 3), for platelets and ablated neutrophils, respectively. For platelets, those positive for Pv and Pf had a daily increase of 0.11 x 10^4^ platelets/mm^3^. Pf positives showed a daily decrease of 0.10% of banded neutrophils, whereas Pv infections had a daily decrease of 0.1% less than that observed for Pf infections.

**Table 3:** Variation over time of the hematological profile related to *Plasmodium* species.

Variable	Model 1					
	Time		*P. vivax*		*Time x P. vivax*	
	Coef.	(95% CI)	Coef.	(95% CI)	Coef.	(95% CI)
Hematocrit (%)	0.00	(-0.06 - 0.07)	0.66	(-0.77 - 2.08)	0.03	(-0.04 - 0.1)
WBC (10³/mm³)	0.01	(-0.01 - 0.02)	-0.11	(-0.46 - 0.24)	0.01	(-0.01 - 0.02)
Banded (%) ^a^	-0.10 ^b^	(-0.13 - -0.07 ^b^)	-0.48 ^b^	(-0.9 - -0.07 ^b^)	-0.06 ^b^	(-0.11 - 0.00^ b^)
Segmented (%)	0.08	(-0.04 - 0.2)	2.68 ^b^	(0.37 - 5.00 b)	-0.10	(-0.24 - 0.04)
Eosinophiles (%)	0.03	(-0.04 - 0.11)	-0.88	(-2.43 - 0.66)	0.08	(-0.02 - 0.17)
Lymphocytes (%)	0.00	(-0.11 - 0.12)	-0.57	(-3.26 - 2.12)	-0.02	(-0.15 - 0.11)
Platelets (10⁴/mm³)	0.11 ^b^	(0.06 - 0.16^ b^)	0.17	(-0.71 - 1.04)	-0.01	(-0.07 - 0.04)

Longitudinal model 1 with interaction term (x) between time (in days) and infecting species (*P. falciparum* as reference), adjusted for age and sex as confounders. This model assumes that time is continuous and the result is linear with respect to time.

Model: Y = b0 + Time + Species + Times*Species + Age + Sex (Time as continuous variable)

WBC: white blood cell count.

a A negative binomial distribution was used due to overdispersion.

b Coefficients with a p-value < 0.05.

With model 2 (Table 4), we found a nonlinear relationship between time and outcome for eosinophils, hematocrit and segmented neutrophils with no difference between *Plasmodium* species. In Pf and Pv infections, eosinophils had an initial mean increase of 5.9% at visit 2, day 7 (during treatment) and a reduced increase of 2.6% at visit 3, day 28 (after treatment) when compared to the initial measurement (visit 1, day 0, before treatment). This is why results show a decline without achieving normalization. In contrast, hematocrit and segmented neutrophils showed an average decrease of 3.2% and 3.1% respectively at visit 2, day 7 (during treatment), but no difference was found at visit 3, day 28 (after treatment) compared to the baseline (visit 1, day 0, before treatment), thus achieving normalization of the values. In this model, only banded neutrophils from Pv-infected participants were different at visit 2 with a 1.9% decrease, lower than in Pf-infected participants, but not at day 28 (after treatment).

**Table 4:** Variation over time of the hematological profile, related to Plasmodium species at day 7 (visit 2) and day 28 (visit 3).

Variable	Model 2									
	Visit 2 day 7		Visit 3 day 28		day 0 Visit 1x*P. vivax*		day 7 Visit 2x*P. vivax*		day 28 Visit 3x*P. vivax*	
	Coef.	(95% CI)	Coef.	(95% CI)	Coef.	(95% CI)	Coef.	(95% CI)	Coef.	(95% CI)
Hematocrit (%)	-3.15 ^b^	(-4.45 - -1.85 ^b^)	-1.38	(-3.13 - 0.37)	0.46	(-1.03 - 1.96)	0.59	(-1.00 - 2.18)	1.11	(-0.84 - 3.06)
RGB (10³/mm³)	0.19	(-0.21 - 0.59)	0.28	(-0.11 - 0.66)	-0.19	(-0.57 - 0.18)	0.25	(-0.19 - 0.70)	0.25	(-0.18 - 0.68)
Banded (%) ^a^	-2.92 ^b^	(-3.76 - -2.09 ^b^)	-2.71 ^b^	(-3.51 -1.91 ^b^)	-0.53 ^b^	(-0.99 -0.08 ^b^)	-1.87 ^b^	(-3.51 - 0.24 ^b^)	-0.59	(-1.67 - 0.50
Segmented (%)	-3.12 ^b^	(-5.97 - -0.27 ^b^)	1.15	(-2.28 - 4.57)	1.94	(-0.90 - 4.77)	1.06	(-2.34 - 4.46)	-2.33	(-6.29 - 1.64)
Eosinophiles (%)	5.88 ^b^	(3.37 - 8.39 ^b^)	2.59 ^b^	(0.52 - 4.66 ^b^)	-0.28	(-2.04 - 1.47)	-0.68	(-3.63 - 2.28)	1.71	(-0.94 - 4.36)
Lymphocytes (%)	1.21	(-1.60 - 4.01)	0.26	(-2.79 - 3.32)	0.04	(-3.02 - 3.11)	-1.70	(-5.05 - 1.64)	-0.82	(-4.49 - 2.85)
Platelets (10⁴/mm³)	1.60 ^b^	(0.45 - 2.75 ^b^)	3.46 ^b^	(2.05 - 4.88 ^b^)	0.04	(-0.99 - 1.06)	0.24	(-1.15 - 1.63)	-0.22	(-1.85 - 1.40)

Longitudinal model 2 with an interaction term (x) between the number of visits (1 day 0 as reference) and the infecting species (*P. falciparum* as reference), adjusted for age and sex as confounders. This model assumes that time is discrete and that the outcome may be nonlinear with respect to time.

Model: Y = b0 + Visit + Species + Visit*Species + Age + Sex (Time as a categorical variable, according to visit).

WBC: white blood cell count.

a A negative binomial distribution was used due to overdispersion.

b Coefficients with a p value < 0.05.

## DISCUSSION

In this study we found that patients infected with Pv and Pf had lower infection indicators such as hematocrit, WBC, eosinophils, and platelets, in contrast, segmented neutrophils and banded neutrophils had higher values. We found variations of the hematological profile over time (before, during and after treatment) in both *Plasmodium* species, with a daily decrease in ablated neutrophils and a daily increase in platelets. In addition, regarding the differences by *Plasmodium* species over time, the Pv-infected participants showed a lower percentage of banded neutrophils compared to the Pf-infected participants.

Our study is one of the first reports in the Loreto region that demonstrates the variations in hematological profiles over time (before, during and after treatment) in patients infected with uncomplicated malaria according to *Plasmodium* species (Pv or Pf), with hematological indicators such as hematocrit, WBC, banded neutrophils, segmented neutrophils, eosinophils, and platelets being statistically different (p < 0.001) from the negative group (participants without malaria). 

The elimination of malaria remains a challenge in malaria endemic countries, because it can be a fatal disease and about half of the world’s population is at risk of contracting it ^2^, which is why, in 2015, WHO launched the Global Technical Strategy for Malaria 2016-2030, highlighting the urgent need to increase interventions, improve preventive measures, diagnostic testing, treatment and surveillance of the disease. In Peru, malaria cases mostly occur in the Amazon, mainly in the Loreto region, with more than 85% of the country’s cases ^5^, and with the presence of the two most dangerous species (Pv and Pf), making it a major public health concern.

Malaria is known to produce hematological abnormalities including anemia and thrombocytopenia due to Pf and Pv, which are considered a hallmark of malaria ^11^ ^,^ ^19^. Hematologic changes are the most common complications encountered in malaria patients and have been correlated with mortality and prognosis in malaria-infected patients ^9^ ^,^ ^19^. Evaluating the white blood cell count and its differentials are among the most basic and primary studies done in patients presenting with fever of short duration. Likewise, neutrophils are essential to fight the disease mainly when the patient presents acute fever, which is expected to decrease after the patient is treated ^20^ ^,^ ^21^.

We found that white blood cell levels were lower in Pv and Pf infected patients, which is similar to other studies conducted in endemic areas ^19^ ^,^ ^22^. Unlike another study in the Amazon (Brazil) ^23^, we found an increase in the segmented neutrophil count in malaria-infected patients compared to controls, although it was not as marked as what was described by Kini and Chandrashekhar ^19^. However, WBC levels were low in infected persons, and did not show leukopenia (<4000 cells/µL), as reported by McKenzie *et al*. ^24^ in a previous study in Iquitos, Peru. It has been described that the WBC count decreases at the same time as fever begins and infection becomes detectable by microscopy ^18^. In addition, lower white blood cell counts in infected persons are characteristic at the onset of a blood infection ^25^.

Likewise, thrombocytopenia is one of the most persistent symptoms in severe malaria and manifests in varying percentages with different severity degrees; it has also been reported as a sensitive marker for the diagnosis of malaria in acute febrile illness ^26^ ^,^ ^27^. In this study thrombocytopenia was found to be associated with *Plasmodium* infections, and was not related to prognostic severity.

Although neutrophil levels were high in Pv infections, banded neutrophils showed higher levels in Pf infections, similar to those reported in studies from India ^28^ where the ratio of counts was uninfected < Pv < Pf. These high levels in Pf infections could be due to the large inflammatory response caused by the parasites. Banded neutrophils are considered acute phase markers with a tendency to increase on the first day of blood infection ^23^.

The high number of eosinophils in the negative group (9%) is common in the Peruvian Amazon and may be explained by the high prevalence of soil-transmitted helminth infections ^29^ and protozoa (47%) ^30^, an aspect to be considered for future studies in order to avoid confounding factors.

It is necessary to recognize some limitations of our study, first the age of the data (2010-2012) and the small number of diagnosed *Plasmodium* infections, which did not allow a broader analysis of the differences in the levels and composition of leukocytes in these two *Plasmodium* species. Another limitation was the absence of a clinical variable such as duration of symptoms, comorbidities (HIV, malnutrition, etc.) or the presence of pregnancy, which are associated with hematological changes. Despite these limitations, this study has allowed us to find that hematological variations do exist in patients infected with *Plasmodium* sp. in the Peruvian Amazon.

In conclusion, malaria infections produced by Pv and Pf, at the onset of infection (before treatment) show a decrease of the hematocrit, eosinophils, and platelets, with an increase in banded neutrophils, showing that these hematological parameters vary over time during and after treatment; platelets increased and banded neutrophils decreased. Differences between *Plasmodium* species over time showed a marked daily decrease in banded neutrophils in Pv-infected patients compared to Pf. These results demonstrate that the hematological profile in uncomplicated malaria-positive participants varies over time during and after treatment, and is an indicator of disease progression that can help in the therapeutic surveillance of *Plasmodium*-infected patients. Future studies should measure hemoglobin levels to avoid confusion and clarify the role of rapid diagnosis for active case detection with the onset of anemia and the study of hematologic profiles in patients who develop severe malaria.
