# Supplementary material for: Hematological profiles of malaria-infected patients in an endemic area of Peru
Source: Rev Peru Med Exp Salud Publica. 2022 Sep 30;39(3):336–44. doi: 10.17843/rpmesp.2022.393.11908 (PMC11397594; doi:10.17843/rpmesp.2022.393.11908)
Supplement: Translation

# Translation: Perfiles hematológicos en pacientes infectados con malaria en un área endémica del Perú

**Authors:** Viviana Pinedo-Cancino, Katty M. Arista, Andree Valle-Campos, Rafael Saavedra-Langer, Cristian Roca, José-Manuel Ramos-Rincón, Maritza Calderón, Oralee H. Branch

**Affiliations:** 1 Laboratorio de Investigación de Productos Naturales Antiparasitarios de la Amazonía, Centro de Investigación de Recursos Naturales, Universidad Nacional de la Amazonía Peruana, Iquitos, Perú.; 2 Facultad de Medicina Humana, Universidad Nacional de la Amazonía Peruana, Iquitos, Perú.; 3 Departamento de Ciencias Biológicas. Universidad Nacional Mayor de San Marcos, Lima, Perú.; 4 Departamento de Bioquímica e Inmunología, Instituto de Ciencias Biológicas, Universidad Federal de Minas Gerais, Belo Horizonte, Minas Gerais, Brazil.; 5 Departamento de Microbiología e Inmunología, Universidad de Carolina del Norte, Chapel Hill, Estados Unidos.; 6 Departamento de Medicina Interna, Hospital General Universitario de Alicante-ISABIAL, Alicante, España.; 7 Departamento de Medicina Clínica, Universidad Miguel Hernández de Elche, Alicante, España.; 8 Laboratorio de Investigación en Enfermedades Infecciosas, Universidad Peruana Cayetano Heredia, Lima, Perú.; 9 Lark Health, Estudios clínicos, Mountain View, California, Estados Unidos.

**Keywords:** *Plasmodium*, Recuento de Células Sanguíneas, Neutrófilos, Plaquetas, Enfermedad Tropical, Amazonía Peruana, Parasitosis

## Abstract

**Objetivos.:**

Evaluar la variación de los perfiles hematológicos antes, durante y después del tratamiento de pacientes infectados con malaria no complicada por *Plasmodium vivax *(Pv) y *P. falciparum *(Pf) en una población de la región Loreto.

**Materiales y métodos.:**

El estudio se realizó entre 2010 y 2012, en Zungarococha (Iquitos). Los 425 participantes tuvieron tres visitas (visita 1-día 0-antes del tratamiento, visita 2-día 7-durante tratamiento, visita 3-día 28-después del tratamiento), hemograma completo, diagnóstico microscópico y molecular (PCR).

**Resultados.:**

En la primera visita, se encontraron 93 (21,9%) positivos a Pv y 34 (8,0%) a Pf. Todos los positivos mostraron una reducción en los indicadores hematológicos de hematocrito, recuento de glóbulos blancos (RGB), neutrófilos abastonados y segmentados, eosinófilos y plaquetas (p<0.001) en comparación con el grupo negativo. Se encontró un porcentaje mayor de neutrófilos abastonados en Pf y de neutrófilos segmentados en Pv comparado al grupo negativo. Se observó variaciones en los perfiles hematológicos después del tratamiento para ambas especies, los neutrófilos abastonados disminuyeron, las plaquetas aumentaron, los eosinófilos se incrementaron al día 7 y decaen el día 28, el hematocrito y los neutrófilos segmentados disminuyeron al día 7 y se normalizaron el día 28. Las diferencias entre especies en el tiempo mostraron una disminución diaria de neutrófilos abastonados en infectados con Pv que en Pf.

**Conclusiones.:**

El perfil hematológico en pacientes positivos a malaria no complicada varía en el tiempo durante y después del tratamiento. Estos son indicadores de la progresión de la enfermedad y ayudan en la vigilancia terapéutica de pacientes infectados con *Plasmodium. *

## INTRODUCCIÓN

La malaria o paludismo ha causado estragos en la humanidad desde la antigüedad y sigue siendo un problema de salud pública de prioridad en regiones tropicales y subtropicales del mundo. En la actualidad son cinco especies de parásitos del género* Plasmodium* que causan enfermedad en los humanos, que se transmiten por la picadura de los mosquitos del género *Anopheles, *siendo *Plasmodium vivax *(Pv) y* Plasmodium falciparum *(Pf) los más peligrosos y frecuentes ^1^.

En 2020 se produjeron en todo el mundo 241 millones de casos de paludismo y 627 000 muertes, con un aumento de 14 millones de casos y 69 000 muertes en relación a las cifras de 2019 ^2^; observándose a nivel mundial un marcado aumento en los casos y muertes por malaria, a pesar de todo el esfuerzo desplegado por los programas de la lucha contra la malaria, para lograr su eliminación en los países endémicos, que se vieron afectados por las interrupciones de los servicios de salud durante la pandemia de la COVID-19 ^3^. 

En Perú, la malaria permanentemente amenaza la salud de la población, principalmente en el departamento de Loreto (capital, Iquitos) donde se concentra el 90% de los casos, con predominio de Pv seguida por Pf ^4^ ^-^ ^6^ *, *clasificado como una región endémica de baja transmisión entre comunidades rurales y periurbanas, aunque existen focos altos de transmisión en algunos lugares de Loreto ^7^. Las características clínicas de la malaria son dependientes de la especie y va desde episodios febriles de corta duración, hasta complicaciones sistémicas severas y muerte ^3^ ^,^ ^8^. 

El cambio en los parámetros leucocitarios es la complicación más común encontrada en pacientes maláricos y cumple un rol importante en la gravedad clínica de la enfermedad y la mortalidad ^9^. La malaria complicada con insuficiencia orgánica grave o anomalías en la sangre generalmente se asocia con Pf ^3^ ^,^ ^10^ y además se reporta anemia grave, existen otras alteraciones como trombocitopenia o reducción en el número de plaquetas, leucocitosis o leucopenia, hemoglobinuria, colapso y choque cardiovascular, lesión renal aguda, acidosis metabólica e hipoglucemia ^11^ ^-^ ^13^. En contraste, la malaria leve con formas benignas se relaciona a Pv, pero también es capaz de producir anemia grave, *shock*, lesión pulmonar, insuficiencia renal aguda y malaria cerebral ^12^ ^,^ ^13^. 

El diagnóstico temprano es importante para un tratamiento rápido y oportuno del manejo de la enfermedad ^1^ ^-^ ^3^. Sin embargo, el conocimiento de las características hematológicas de la malaria se limita a Pf de regiones del África y Asia ^14^ ^,^ ^15^, y poco o nada se conoce de las características leucocitarias de Pv en poblaciones endémicas de Loreto, su relación como diagnóstico hematológico podría permitir un manejo más adecuado de las posibles complicaciones de la malaria. El presente trabajo, evaluó la variación de los perfiles hematológicos antes, durante y después del tratamiento de pacientes infectados con malaria no complicada por *Plasmodium vivax *(Pv) y *P. falciparum *(Pf) en una población de la región Loreto, comparándolo con individuos sanos de la misma área para su potencial uso como predictores de progresión de la enfermedad y eficacia del tratamiento.

**MENSAJE CLAVE**

Motivación para realizar el estudio: conocer si existen variaciones hematológicas durante la infección por malaria no complicada antes, durante y después del tratamiento.

Principales hallazgos: variaciones en los perfiles hematológicos ocurre en pacientes infectados con Plasmodium, que se van normalizando con el tiempo después del tratamiento y que se diferencia por especie.

Implicancias: la predicción de estas variaciones permitiría establecer una temprana intervención terapéutica con el fin de prevenir la ocurrencia de complicaciones.

## MATERIALES Y MÉTODOS

### Tipo de estudio, población de estudio y colección de datos

Se realizó un estudio descriptivo longitudinal con muestras colectadas entre el 2010 y 2012, de un estudio de cohorte del Proyecto Inmunología y Genética de la Malaria en la Amazonía, desarrollada en la comunidad de Zungarococha, que agrupa a cuatro pueblos: Zungarocoha, Puerto Almendra, Nina Rumi y Llanchama (Iquitos, Perú), donde las infecciones por Pv y Pf son frecuentemente asintomáticas con baja densidad parasitaria ^16^. Se incluyeron en el estudio los participantes que ingresaron al proyecto y que contaron con consentimiento y/o asentimiento firmado y que aceptaron el uso futuro de sus muestras, fueron excluidos todos los pacientes que viajaron en el transcurso de las visitas. Todos los participantes tuvieron tres visitas: visita 1-día 0 (antes del tratamiento), visita 2-día 7 (durante tratamiento), visita 3-día 28 (después del tratamiento).

El proceso de recolección de muestra, se dio tanto por búsqueda pasiva como activa, según se detalla en Branch, *et al*. ^16^. Brevemente: por cada participante se obtuvo una muestra de sangre a través de un pinchazo en el dedo colectando 500 µL en microtubos de 1,5 mL con 40 µL de EDTA al 0,05 M o punción venosa usando tubos Vacutainer de 3 mL con EDTA K2. Para el hematocrito se llenó los tubos capilares de 75 mm de longitud y unos 1,5 mm de diámetro, además, se realizó el extendido sanguíneo en 2 láminas para el hemograma y el diagnóstico microscópico de malaria. Los eritrocitos se separaron del plasma por centrifugación a 1200 rpm durante 10 min y se congelaron a -20 ºC hasta la extracción del ADN. 

El análisis hematológico se realizó de forma manual, el hematocrito se midió como volumen de las células empaquetadas después de la centrifugación a 1200 rpm por 5 min, empleando una regla milimétrica que midió las columnas formadas en el interior de los capilares y se calculó el porcentaje que le corresponde a la columna de eritrocitos, con una regla de tres. El frotis fue coloreado con solución Wright por un minuto a temperatura ambiente. Posteriormente, se adicionó agua en partes iguales al colorante y se dejó actuar por 10 min, se lavó, se dejó secar para luego realizar en el microscopio, con el objetivo de inmersión (100x), el recuento diferencial de glóbulos blancos hasta 100 células. Los técnicos que trabajaron en el análisis hematológico de los frotis fueron diferentes y solo conocían los resultados de las muestras que analizaron.

El frotis sanguíneo para el diagnóstico de malaria fue coloreado con tinción Giemsa y observado bajo el microscopio con aceite de inmersión; para establecer la densidad parasitaria se usó el método de cruces y el método de parásitos por microlitro de sangre ^16^.

El ADN de *Plasmodium *fue extraído a partir de 200 µL de eritrocitos usando el minikit para sangre QIAamp DNA (Qiagen, Hilden, Germany). Seguidamente, las muestras de ADN se conservaron a -20 ºC hasta la amplificación. Se empleó para el diagnóstico molecular la técnica semi *nested multiplex malaria* PCR (SnM-PCR) de forma convencional, con una amplificación externa e interna, y electroforesis con gel de agarosa al 1,5 % para la identificación de las especies de *Plasmodium* ^17^. 

### Análisis estadístico

El análisis de los datos se realizó con el paquete estadístico Stata v.15.0 (StataCorp, College Station, TX, USA). Para las comparaciones de características demográficas y de laboratorio entre el grupo de participantes positivos a *Plasmodium* sp y el grupo de participantes negativos (participantes sin malaria) se utilizó las pruebas de Chi cuadrado y Kruskal-Wallis, con un intervalo de confianza de 95%, siendo una p significativa <0,05. Se eligió la prueba de Chi cuadrado para poner a prueba las hipótesis de igualdad de proporciones de las variables categóricas, y la prueba Kruskal-Wallis para poner a prueba las hipótesis de igualdad de medianas en las variables numéricas continuas con distribución no normal. 

Los criterios para construir los modelos lineales fueron los siguientes: Todas las variables del perfil hematológico fueron incluidas en un modelo lineal generalizado con enlace de identidad ajustado por edad y sexo, para cuantificar las variaciones de los perfiles hematológicos de cada especie de *Plasmodium *versus el grupo negativo, en la visita 1 (correspondiente al día 0 del estudio). Se interpretó los coeficientes del modelo como el promedio de la magnitud de cambio, en su respectiva unidad de medida, del indicador hematológico en una determinada especie de *Plasmodium* con respecto al grupo negativo. El análisis de las variaciones en el perfil hematológico en el tiempo: visita 2-día 7 (durante tratamiento) y visita 3-día 28 (después del tratamiento) con la especie de *Plasmodium, *utilizamos un modelo de regresión lineal con distribución Gaussian y función con enlace de identidad para cada variable hematológica como resultado. Se insertó el tiempo en dos modelos separados: i) asumiendo linealidad de tiempo-resultado y ii) día de cada visita. Modelo 1, se consideró el tiempo como una variable continua, usando el intervalo de tiempo en días, entre la medición inicial (visita 1-día 0, antes del tratamiento) y la fecha de seguimiento (visita 2-día 7, durante el tratamiento y visita 3-día 28, después del tratamiento). Modelo 2, se consideró el tiempo como una variable categórica según visita, donde la medición inicial fue el día 0 (visita 1), el día 7 (visita 2) y el día 28 (visita 3). Cada participante tenía tres mediciones para cada variable hematológica. La exposición invariable en el tiempo fue la especie de *Plasmodium*, y se ajustó por edad y sexo. El análisis está disponible en GitHub (https://github.com/avallecam/hemogr/) y en Zenodo (https://doi.org/10.5281/zenodo. 4014204). 

### Aspectos éticos

Toda la investigación fue ejecutada de acuerdo con los principios expresados en la Declaración de Helsinki y la participación fue voluntaria. Fue aprobado por el Comité de Ética de la Universidad de New York (NYU Institional Review Board [IRB] APROVAL NO. 08-982) y del Ministerio de Salud peruano y el Comité de Ética del Instituto Nacional de Salud. Luego de recibir información del estudio, los individuos voluntarios a participar firmaron un consentimiento informado.

## RESULTADOS

Entre los años 2010 a 2012 fueron evaluados 425 participantes con un rango de edad entre 10 y 44 años, 236 correspondieron al sexo femenino y 189 al sexo masculino. 

A todos los participantes se confirmó el diagnóstico de malaria por microscopía, que resultó en 93 (21,9%) positivos a Pv, 34 (8,0%) positivos a Pf y 298 (70,1%) negativos para malaria. Todos los resultados positivos por microscopía y 99 participantes seleccionados aleatoriamente de los 298 negativos por microscopía fueron evaluados por PCR para confirmar los resultados. 

Como se muestra en la Tabla 1, los indicadores hematológicos de medición inicial como hematocrito, recuento de glóbulo sanguíneas (RGB), neutrófilos abastonados, neutrófilos segmentados, eosinófilos y plaquetas fueron estadísticamente diferentes (p < 0,001) entre participantes infectados con malaria no complicada comparado al grupo negativo (participantes sin malaria). A diferencia de los monocitos y linfocitos en los grupos de estudio no fueron estadísticamente diferentes entre sí.

**Tabla 1:** Distribución inicial (correspondiente a la visita 1 en el día 0 del estudio) de las características demográficas y el perfil hematológico entre participantes negativos y participantes positivos por especie de *Plasmodium.*

Variable	Negativo		*P. vivax*		*P. falciparum*		Valor de p ^b^
	N=298		N=93		N=34		
	Mediana (RIC)		Mediana (RIC)		Mediana (RIC)		
Edad (años)	28	(17,0 - 42,8)	17	(10,0 - 39,0)	24,5	(14,2 - 44,2)	0,001
Sexo^a^							0,082 ^c^
Mujer	175	(58,7)	47	(50,5)	14	(41,2)	
Hombre	123	(41,3)	46	(49,5)	20	(58,8)	
Hematocrito (%)	42	(39,0 - 44,0)	39	(36,0 - 42,0)	39,5	(37,2 - 41,8)	<0,001
Leucocitos (10³/mm³)	6.2	(5,9 - 7,0)	5.8	(5,1 - 6,0)	5.9	(5,0 - 6,2)	<0,001
Abastonados (%)	0	(0,0 - 0,0)	2	(0,0 - 4,0)	4.5	(0,0 - 7,5)	<0,001
Neutrófilos (%)	58	(50,2 - 60,0)	60	(56,0 - 62,0)	57,5	(54,0 - 60,0)	0,009
Eosinófilos (%)	9	(5,0 - 12,0)	5	(3,0 - 8,0)	5	(4,0 - 8,8)	<0,001
Linfocitos (%)	34	(29,2 - 38,8)	32	(30,0 - 35,0)	30	(27,0 - 36,0)	0,075
Plaquetas (10⁴/mm³)	17.3	(16,0 - 19,7)	14.9	(13,6 - 17,1)	14.8	(13,2 - 17,4)	<0,001
Monocitos (%) ^a^							0,283 ^c^
0	289	(97,0)	91	(97,8)	32	(94,1)	
1	5	(1,7)	2	(2,2)	0	(0,0)	
2	3	(1,0)	0	(0,0)	2	(5,9)	
3	1	(0,3)	0	(0,0)	0	(0,0)	
Basófilos (%) ^a^							0,159 ^c^
0	279	(93,6)	93	(100,0)	34	(100,0)	
1	15	(5,0)	0	(0,0)	0	(0,0)	
2	3	(1,0)	0	(0,0)	0	(0,0)	
3	1	(0,3)	0	(0,0)	0	(0,0)	

RIC: rango intercuartil.

a Número y porcentaje, N (%).

b Prueba de Kruskal-Wallis

c Prueba de Chi cuadrado

Para la visita 1 (día 0 del estudio), los análisis del modelo lineal generalizado con enlace de identidad ajustado por edad y género realizado para cuantificar las diferencias entre los participantes infectados con *P. vivax* y *P. falciparum* (Figura 1) comparado con el grupo negativo (Tabla 2), demostró que los participantes infectados con Pv y Pf obtuvieron bajos porcentajes para hematocrito (Pv: -2,3%, Pf: -2,7%), RGB (Pv: -0,6%, Pf: -0,5%), eosinófilos (Pv: -3,6%, Pf: -3,2%) y plaquetas (Pv: -2,8%, Pf: -2,6% x 10^4^ plaquetas/mm^3^) en comparación con el grupo negativo (participantes sin malaria). Al contrario, los participantes infectados con malaria obtuvieron altos porcentajes de neutrófilos abastonados principalmente Pf (Pv: 2,1%, Pf: 3,7%) comparado con el grupo negativo. Un patrón diferente fue observado solo en los neutrófilos segmentados, donde únicamente los infectados con Pv tuvieron un porcentaje más alto (2,7%) comparado a Pf y el grupo negativo.

**Figura 1:** Perfiles hematológicos por día de visita y por especie de *Plasmodium.*

**Tabla 2:** Diferencias hematológicas en la visita 1 (día 0 del estudio) entre participantes negativos y positivos por especie de Plasmodium en un análisis de regresión múltiple.

Variable	Negativo		*P. vivax*			*P. falciparum*		
	Mediana	(RIC)	Coef.	(IC 95%)	Valor p	Coef.	(IC 95%)	Valor de p
Hematocrito (%)	42,0	(39,0 - 44,0]	-2,26	(-3,38 - -1,13)	<0,001	-2,71	(-4,36 - -1,06)	0,001
RGB (10³/mm³)	6,2	(5,9 - 7,0)	-0,64	(-0,84 - -0,44)	<0,001	-0,46	(-0,78 - -0,15)	0,004
Abastonados (%)	0,0	(0,0 - 0,0)	2,11	(1,64 - 2,59)	<0,001	3,70	(3,05 - 4,34)	<0,001
Segmentados (%)	58,0	(50,2 - 60,0)	2,71	(1,00 - 4,43)	0,002	0,75	(-1,83 - 3,33)	0,568
Eosinófilos (%)	9,0	(5,0 - 12,0)	-3,58	(-4,97 - -2,19)	<0,001	-3,21	(-5,37 - -1,04)	0,004
Linfocitos (%)	34,0	(29,2 - 38,8)	-1,13	(-2,93 - 0,68)	0,221	-1,22	(-4,02 - 1,57)	0,391
Plaquetas (10⁴/mm³)	17,3	(16,0 - 19,7)	-2,77	(-3,48 - -2,07)	<0,001	-2,62	(-3,72 - -1,52)	<0,001
Monocitos (%)	0,0	(0,0 - 0,0)	-0,04	(-0,10 - 0,03)	0,264	0,06	(-0,05 - 0,18)	0,274
Basófilos (%)	0,0	(0,0 - 0,0)	-0,08	(-0,15 - -0,01)	0,026	-0,08	(-0,20 - 0,03)	0,170

RIC: rango intercuartil, RGB: recuento de glóbulos blancos

Todos los coeficientes de las especies de *Plasmodium *se calcularon en comparación con el grupo de participantes negativos y participantes positivos por especie de *Plasmodium*

El análisis de las variaciones en el perfil hematológico en el tiempo compara la visita 2-día 7 (durante tratamiento) y la visita 3-día 28 (después del tratamiento) con la visita 1-día 0 y la especie de *Plasmodium*. Se encontró en el modelo 1 (Tabla 3) una relación lineal continua de incremento y disminución entre el tiempo y el resultado para las plaquetas y los neutrófilos abastonados, respectivamente. Para las plaquetas, los positivos a Pv y Pf tuvieron un incremento diario de 0,11 x 10^4^ plaquetas/mm^3^. Para los neutrófilos abastonados, los positivos a Pf tuvieron una disminución diaria del 0,10%, mientras que las infecciones por Pv tuvieron una disminución diaria de 0,1% menor a la observada en las infecciones por Pf.

**Tabla 3:** Variación en el tiempo del perfil hematológico relacionado con la especie de *Plasmodium.*

Variable	Modelo 1					
	Tiempo		*P. vivax*		*Tiempo x P. vivax*	
	Coef.	(IC 95%)	Coef.	(IC 95%)	Coef.	(IC 95%)
Hematocrito (%)	0,00	(-0,06 - 0,07)	0,66	(-0,77 - 2.08)	0,03	(-0,04 - 0,1)
RGB (10³/mm³)	0,01	(-0,01 - 0,02)	-0,11	(-0,46 - 0.24)	0,01	(-0,01 - 0,02)
Abastonados (%) ^a^	-0,10 ^b^	(-0,13 - -0,07 ^b^)	-0,48 ^b^	(-0,9 - -0.07 b)	-0,06 ^b^	(-0,11 - 0,00^ b^)
Segmentados (%)	0,08	(-0,04 - 0,2)	2,68 ^b^	(0,37 - 5.00 b)	-0,10	(-0,24 - 0,04)
Eosinófilos (%)	0,03	(-0,04 - 0,11)	-0,88	(-2,43 - 0.66)	0,08	(-0,02 - 0,17)
Linfocitos (%)	0,00	(-0,11 - 0,12)	-0,57	(-3,26 - 2.12)	-0,02	(-0,15 - 0,11)
Plaquetas (10⁴/mm³)	0,11 ^b^	(0,06 - 0,16^ b^)	0,17	(-0,71 - 1.04)	-0,01	(-0,07 - 0,04)

Modelo longitudinal 1 con término de interacción (x) entre el tiempo (en días) y la especie de infección (*P. falciparum* como referencia), ajustado por edad y sexo como confusores. Este modelo asume que el tiempo es continuo y el resultado es lineal con respecto al tiempo.

Modelo: Y = b0 + Tiempo + Especie + Tiempos*Especie + Edad + Sexo (Tiempo como variable continua)

RGB: recuento de glóbulos blancos

a Se utilizó una distribución binomial negativa debido a la sobredispersión.

b Coeficientes con un valor de p < 0,05.

Con el modelo 2 (Tabla 4), se encontró una relación no lineal entre tiempo y resultado para eosinófilos, hematocrito y neutrófilos segmentados sin diferencia entre especies de *Plasmodium*. En infecciones por Pf y Pv, los eosinófilos tuvieron un aumento promedio inicial del 5,9% en la visita 2-día 7 (durante el tratamiento) y una reducción de su aumento a 2,6% en la visita 3-día 28 (después del tratamiento) con respecto a la medición inicial (visita 1-día 0, antes del tratamiento). Por ello es que se observa un decaimiento sin lograr la normalización. Por el contrario, el hematocrito y los neutrófilos segmentados presentaron una disminución promedio de 3,2% y 3,1% respectivamente en la visita 2-día 7 (durante el tratamiento), pero no se encontró diferencia en la visita 3-día 28 (después del tratamiento) con respecto al basal (visita 1-día 0, antes del tratamiento), lográndose así una normalización de los valores. En este modelo, sólo los neutrófilos abastonados de participantes infectados con Pv fueron diferentes en la visita 2-día 7 con una disminución de 1,9% menor a las observadas en los infectados con Pf, pero no en el día 28 (después del tratamiento).

**Tabla 4:** Variación en el tiempo del perfil hematológico, relacionado con la especie de *Plasmodium *al día 7 (visita 2) y día 28 (visita 3).

Variable	Modelo 2									
	Visita 2 día 7		Visita 3 día 28		día 0 Visita 1x*P. vivax*		día 7 Visita 2x*P. vivax*		día 28 Visita 3x*P. vivax*	
	Coef.	(IC 95%)	Coef.	(IC 95%)	Coef.	(IC 95%)	Coef.	(IC 95%)	Coef	(IC 95%)
Hematocrito (%)	-3,15 ^b^	(-4,45 - -1,85 ^b^)	-1,38	(-3,13 - 0,37)	0,46	(-1,03 - 1,96)	0,59	(-1,00 - 2,18)	1,11	(-0,84 - 3,06)
RGB (10³/mm³)	0,19	(-0,21 - 0,59)	0,28	(-0,11 - 0,66)	-0,19	(-0,57 - 0,18)	0,25	(-0,19 - 0,70)	0,25	(-0,18 - 0,68)
Abastonados (%) ^a^	-2,92 ^b^	(-3,76 - -2,09 ^b^)	-2,71 ^b^	(-3,51 -1,91 ^b^)	-0,53 ^b^	(-0,99 -0,08 ^b^)	-1,87 ^b^	(-3,51 - 0,24 ^b^)	-0,59	(-1,67 - 0,50
Segmentados (%)	-3,12 ^b^	(-5,97 - -0,27 ^b^)	1,15	(-2,28 - 4,57)	1,94	(-0,90 - 4,77)	1,06	(-2,34 - 4,46)	-2,33	(-6,29 - 1,64)
Eosinófilos (%)	5,88 ^b^	(3,37 - 8,39 ^b^)	2,59 ^b^	(0,52 - 4,66 ^b^)	-0,28	(-2,04 - 1,47)	-0,68	(-3,63 - 2,28)	1,71	(-0,94 - 4,36)
Linfocitos (%)	1,21	(-1,60 - 4,01)	0,26	(-2,79 - 3,32)	0,04	(-3,02 - 3,11)	-1,70	(-5,05 - 1,64)	-0,82	(-4,49 - 2,85)
Plaquetas (10⁴/mm³)	1,60 ^b^	(0,45 - 2,75 ^b^)	3,46 ^b^	(2,05 - 4,88 ^b^)	0,04	(-0,99 - 1,06)	0,24	(-1,15 - 1,63)	-0,22	(-1,85 - 1,40)

Modelo longitudinal 2 con un término de interacción (x) entre el número de visitas (1 dia 0 como referencia) y la especie de infección (P. falciparum como referencia), ajustado por la edad y el sexo como confusores. Este modelo asume que el tiempo es discreto y que el resultado puede ser no lineal con respecto al tiempo.

Modelo: Y = b0 + Visita + Especie + Visita*Especie + Edad + Sexo (Tiempo como variable categórica, según visita)

RGB: recuento de glóbulos blancos

a Se utilizó una distribución binomial negativa debido a la sobredispersión.

b Coeficientes con un valor de p < 0,05.

## DISCUSIÓN

En el presente estudio se encontró que los pacientes infectados con Pv y Pf tienen los indicadores de infección como hematocrito, RGB, eosinófilos y plaquetas valores más bajos, en contraste, con los neutrófilos segmentados y los neutrófilos abastonados que tuvieron valores más altos. En ambas especies de *Plasmodium *se observó variaciones en el perfil hematológico en el tiempo (antes, durante y después del tratamiento) con una disminución diaria de los neutrófilos abastonados y un aumento diario de las plaquetas. Además, en las diferencias por especie de *Plasmodium* en el tiempo, fueron los participantes infectados con Pv quienes mostraron un menor porcentaje de neutrófilos abastonados comparados con los participantes infectados con Pf.

Los resultados del presente trabajo serían uno de los primeros reportes para la región Loreto, que demuestran que existen variaciones de los perfiles hematológicos en el tiempo (antes, durante y después del tratamiento) en pacientes infectados con malaria no complicada según especie de *Plasmodium* (Pv o Pf), siendo los indicadores hematológicos como hematocrito, RGB, neutrófilos abastonados, neutrófilos segmentados, eosinófilos y plaquetas que se diferenciaron estadísticamente (p < 0,001) del grupo negativo (participantes sin malaria). 

La eliminación de la malaria (o paludismo) sigue siendo un desafío en los países endémicos que la padecen, porque es una enfermedad que puede ser mortal y cerca de la mitad de la población del mundo corre el riesgo de contraerla ^2^, razón por la cual, la OMS en 2015 lanzó la *Estrategia técnica mundial contra la malaria 2016-2030*, destacando la urgente necesidad de aumentar la intervenciones, mejorar las medidas preventivas, las pruebas de diagnóstico, el tratamiento y la vigilancia de la enfermedad. En Perú, la malaria se concentra en el llano amazónico, principalmente en la región Loreto, con más del 85% de los casos del país ^5^, con presencia de las dos especies más peligrosas Pv y Pf, por eso es un importante problema de salud pública. 

Es conocido que la malaria produce anomalías hematológicas que incluye la anemia y la trombocitopenia por Pf y Pv, que se consideran como un sello distintivo de la malaria ^11^ ^,^ ^19^. Los cambios hematológicos son las complicaciones más comunes encontradas en pacientes palúdicos la cual ha estado correlacionada con la mortalidad y el pronóstico de pacientes infectados con malaria ^9^ ^,^ ^19^. El recuento de glóbulos blancos y sus diferenciales están entre las más básicas y primarias investigaciones hechas en pacientes que presentaron fiebre de corta duración. Igualmente, los neutrófilos son esenciales para combatir la enfermedad principalmente cuando el paciente presenta fiebre aguda y se espera que esta disminuya luego que el paciente sea tratado ^20^ ^,^ ^21^. 

En el presente estudio, los niveles de glóbulos blancos fueron menores en infectados por Pv y Pf en concordancia con otros estudios desarrollados en áreas endémicas ^19^ ^,^ ^22^. Diferente a otro estudio en la Amazonía (Brasil) ^23^, un aumento en el conteo de neutrófilos segmentados fue observado en pacientes infectados con malaria comparado con los controles, aunque no estaba tan marcado como el descrito por Kini y Chandrashekhar ^19^. Sin embargo, los niveles de glóbulos blancos fueron bajos en las personas infectadas, y no demostraron leucopenia (<4000 células/µL), tal como lo reportó McKenzie* et al.* ^24^ ^) ^en un estudio previo en Iquitos, Perú.Ha sido descrito que el conteo de glóbulos blancos disminuye al mismo tiempo que la fiebre comienza y la infección se convierte en detectable por microscopía ^18^. Además, el conteo menor de glóbulos blancos en personas infectadas son características al inicio de una infección sanguínea ^25^. 

Igualmente, la trombocitopenia es uno de los factores más persistentes en malaria severa y se manifiesta en porcentajes variables y con diferente severidad en los casos de malaria y además ha sido reportado como un marcador sensible para el diagnóstico de malaria en enfermedad febril aguda ^26^ ^,^ ^27^. En este estudio se encontró que la trombocitopenia estaba asociada con las infecciones de* Plasmodium*, y no estaba relacionada a un pronóstico de gravedad. 

Aunque los niveles de neutrófilos fueron elevados en las infecciones por Pv, los neutrófilos abastonados demostraron niveles mayores en Pf, similar a los que fueron reportados en estudios de la India ^28^ donde la relación de los conteos fue no infectados *< *Pv *< *Pf. Estos niveles elevados en infecciones por Pf podrían deberse a la gran respuesta inflamatoria generada por estos parásitos. Los neutrófilos abastonados son considerados marcadores de la fase aguda con tendencia a aumentar en el primer día de la infección sanguínea ^23^. 

El elevado número de eosinófilos en el grupo de negativos (9%) es una circunstancia habitual en la Amazonía peruana y puede ser explicada por la alta prevalencia de infecciones de helmintos transmitidos por el suelo ^29^ y protozoos (47%) ^30^, aspecto a considerar para futuros estudios y así evitar factores de confusión. 

Es necesario reconocer algunas limitaciones de este trabajo, primero la antigüedad de los datos (2010-2012), el escaso número de infecciones por* Plasmodium* diagnosticadas que no permitió analizar con mayor amplitud las diferencias en los niveles y composición de leucocitos en estas dos especies de *Plasmodium*. Otra limitación fue la ausencia de una variable clínica como la duración de los síntomas, comorbilidades (VIH, desnutrición, etc.) o la presencia de embarazo, que se asocian con cambios hematológicos. A pesar de estas limitaciones, este estudio ha permitido observar que sí existen variaciones hematológicas en pacientes infectados con *Plasmodium *sp. en la Amazonía peruana.

En conclusión, las infecciones por malaria producidas por Pv y Pf, al inicio de la infección (antes del tratamiento) muestran una reducción del hematocrito, eosinófilos y plaquetas, con un aumento de neutrófilos abastonados, lo cual demuestra que estos parámetros hematológicos varían en el tiempo durante y después del tratamiento; en el caso de las plaquetas, estas se incrementaron y los neutrófilos abastonados disminuyeron. Las diferencias entre especies de *Plasmodium* en el tiempo mostraron una marcada disminución diaria de neutrófilos abastonados en infectados con Pv en comparación con Pf. Estos resultados demuestran que el perfil hematológico en participantes positivos a malaria no complicada varía en el tiempo durante y después del tratamiento, que son indicadores de la progresión de la enfermedad y ayudan en la vigilancia terapéutica de pacientes infectados con *Plasmodium. *Futuros estudios deberían medir los niveles de hemoglobina para evitar confusiones y esclarecer el papel del diagnóstico rápido para la detección activa de casos con la aparición de anemia y el estudio de perfiles hematológicos en pacientes que desarrollan malaria grave.
